# The ARCA Registry: A Collaborative Global Platform for Advancing Trial Readiness in Autosomal Recessive Cerebellar Ataxias

**DOI:** 10.3389/fneur.2021.677551

**Published:** 2021-06-25

**Authors:** Andreas Traschütz, Selina Reich, Astrid D. Adarmes, Mathieu Anheim, Mahmoud Reza Ashrafi, Jonathan Baets, A. Nazli Basak, Enrico Bertini, Bernard Brais, Cynthia Gagnon, Janina Gburek-Augustat, Hasmet A. Hanagasi, Anna Heinzmann, Rita Horvath, Peter de Jonghe, Christoph Kamm, Peter Klivenyi, Thomas Klopstock, Martina Minnerop, Alexander Münchau, Mathilde Renaud, Richard H. Roxburgh, Filippo M. Santorelli, Tommaso Schirinzi, Deborah A. Sival, Dagmar Timmann, Stefan Vielhaber, Michael Wallner, Bart P. van de Warrenburg, Ginevra Zanni, Stephan Zuchner, Thomas Klockgether, Rebecca Schüle, Ludger Schöls, PREPARE Consortium, Matthis Synofzik

**Affiliations:** ^1^Department of Neurodegenerative Diseases, Hertie-Institute for Clinical Brain Research and Center of Neurology, University of Tübingen, Tübingen, Germany; ^2^German Center for Neurodegenerative Diseases (DZNE), University of Tübingen, Tübingen, Germany; ^3^Unidad de Trastornos del Movimiento, Servicio de Neurología y Neurofisiología Clínica, Instituto de Biomedicina de Sevilla, Hospital Universitario Virgen del Rocío/CSIC/Universidad de Sevilla, Seville, Spain; ^4^Centro de Investigación Biomédica en Red Sobre Enfermedades Neurodegenerativas, Madrid, Spain; ^5^Service de Neurologie, Hôpitaux Universitaires de Strasbourg, Hôpital de Hautepierre, Strasbourg, France; ^6^Fédération de Médecine Translationnelle de Strasbourg, Université de Strasbourg, Strasbourg, France; ^7^Institut de Génétique et de Biologie Moléculaire et Cellulaire, INSERM-U964/CNRS-UMR7104/Université de Strasbourg, Illkirch, France; ^8^Department of Pediatric Neurology, Ataxia Clinic, Growth and Development Research Center, Children's Medical Center, Pediatrics Center of Excellence, Tehran University of Medical Sciences, Tehran, Iran; ^9^Translational Neurosciences, Faculty of Medicine and Health Sciences, UAntwerpen, Antwerp, Belgium; ^10^Laboratory of Neuromuscular Pathology, Institute Born-Bunge, University of Antwerp, Antwerp, Belgium; ^11^Department of Neurology, Neuromuscular Reference Centre, Antwerp University Hospital, Antwerp, Belgium; ^12^Neurodegeneration Research Laboratory, Suna and Inan Kiraç Foundation, KUTTAM, Koç University School of Medicine, Istanbul, Turkey; ^13^Unit of Neuromuscular and Neurodegenerative Diseases, Department of Neurosciences, Bambino Gesù Children's Hospital, IRCCS, Rome, Italy; ^14^Department of Neurology, McGill University, Montreal Neurological Institute, Montréal, QC, Canada; ^15^Centre de Recherche Charles-Le Moyne-Saguenay-Lac-Saint-Jean sur les Innovations en Santé, Sherbrooke University, Sherbrooke, QC, Canada; ^16^Division of Neuropaediatrics, Hospital for Children and Adolescents, University of Leipzig, Leipzig, Germany; ^17^Behavioral Neurology and Movement Disorders Unit, Department of Neurology, Istanbul Faculty of Medicine, Istanbul University, Istanbul, Turkey; ^18^AP-HP, Department of Genetics, Pitié-Salpêtrière University Hospital, Paris, France; ^19^Department of Clinical Neurosciences, University of Cambridge, Cambridge, United Kingdom; ^20^Department of Neurology, University of Rostock, Rostock, Germany; ^21^Department of Neurology, Faculty of Medicine, Albert Szent-Györgyi Clinical Center, Interdisciplinary Excellence Centre, University of Szeged, Szeged, Hungary; ^22^Department of Neurology, Friedrich-Baur-Institute, Ludwig-Maximilians-University of Munich, Munich, Germany; ^23^German Center for Neurodegenerative Diseases (DZNE), Munich, Germany; ^24^Munich Cluster for Systems Neurology (SyNergy), Munich, Germany; ^25^Institute of Neuroscience and Medicine (INM-1), Research Centre Juelich, Juelich, Germany; ^26^Department of Neurology, Center for Movement Disorders and Neuromodulation, Medical Faculty, Heinrich Heine University, Düsseldorf, Germany; ^27^Institute of Clinical Neuroscience and Medical Psychology, Medical Faculty, Heinrich-Heine University, Düsseldorf, Germany; ^28^Neurogenetics, Institute of Systems Motor Science, University of Lübeck, Lübeck, Germany; ^29^Service de Génétique Clinique, CHRU de Nancy, Nancy, France; ^30^INSERM-U1256 NGERE, Université de Lorraine, Nancy, France; ^31^Auckland District Health Board, Auckland, New Zealand; ^32^Centre of Brain Research Neurogenetics Research Clinic, University of Auckland, Auckland, New Zealand; ^33^IRCCS Fondazione Stella Maris, Pisa, Italy; ^34^Neurorehabilitation Unit, Department of Neurosciences, IRCCS Bambino Gesù Children Hospital, Rome, Italy; ^35^Department of Systems Medicine, University of Roma Tor Vergata, Rome, Italy; ^36^Department of Pediatrics, Beatrix Children's Hospital, University Medical Center Groningen, Groningen, Netherlands; ^37^Department of Neurology, Essen University Hospital, University of Duisburg-Essen, Essen, Germany; ^38^Department of Neurology, Otto-von-Guericke University, Magdeburg, Germany; ^39^German Center for Neurodegenerative Diseases (DZNE) Within the Helmholtz Association, Magdeburg, Germany; ^40^Center for Behavioral Brain Sciences, Magdeburg, Germany; ^41^2mt Software GmbH, Ulm, Germany; ^42^Department of Neurology, Donders Institute for Brain, Cognition and Behaviour, Radboud University Medical Centre, Nijmegen, Netherlands; ^43^Dr. John T. Macdonald Foundation Department of Human Genetics and John P. Hussman Institute for Human Genomics, University of Miami Miller School of Medicine, Miami, FL, United States; ^44^Department of Neurology, University Hospital Bonn, Bonn, Germany; ^45^German Center for Neurodegenerative Diseases (DZNE), Bonn, Germany

**Keywords:** ataxia, registry, network, natural history, trial readiness

## Abstract

Autosomal recessive cerebellar ataxias (ARCAs) form an ultrarare yet expanding group of neurodegenerative multisystemic diseases affecting the cerebellum and other neurological or non-neurological systems. With the advent of targeted therapies for ARCAs, disease registries have become a precious source of real-world quantitative and qualitative data complementing knowledge from preclinical studies and clinical trials. Here, we review the *ARCA Registry*, a global collaborative multicenter platform (>15 countries, >30 sites) with the overarching goal to advance trial readiness in ARCAs. It presents a good clinical practice (GCP)- and general data protection regulation (GDPR)-compliant professional-reported registry for multicenter web-based capture of cross-center standardized longitudinal data. Modular electronic case report forms (eCRFs) with core, extended, and optional datasets allow data capture tailored to the participating site's variable interests and resources. The eCRFs cover all key data elements required by regulatory authorities [European Medicines Agency (EMA)] and the European Rare Disease (ERD) platform. They capture genotype, phenotype, and progression and include demographic data, biomarkers, comorbidity, medication, magnetic resonance imaging (MRI), and longitudinal clinician- or patient-reported ratings of ataxia severity, non-ataxia features, disease stage, activities of daily living, and (mental) health status. Moreover, they are aligned to major autosomal-dominant spinocerebellar ataxia (SCA) and sporadic ataxia (SPORTAX) registries in the field, thus allowing for joint and comparative analyses not only across ARCAs but also with SCAs and sporadic ataxias. The registry is at the core of a systematic multi-component ARCA database cluster with a linked biobank and an evolving study database for digital outcome measures. Currently, the registry contains more than 800 patients with almost 1,500 visits representing all ages and disease stages; 65% of patients with established genetic diagnoses capture all the main ARCA genes, and 35% with unsolved diagnoses are targets for advanced next-generation sequencing. The ARCA Registry serves as the backbone of many major European and transatlantic consortia, such as PREPARE, PROSPAX, and the Ataxia Global Initiative, with additional data input from SPORTAX. It has thus become the largest global trial-readiness registry in the ARCA field.

## ARCA Registry: The Overarching Goal

Autosomal recessive cerebellar ataxias (ARCAs) are a heterogeneous group of ultrarare multisystemic neurodegenerative diseases affecting the cerebellum and/or its afferent tracts, often accompanied by damage to other neurological (e.g., corticospinal tract, basal ganglia, vestibular system, and peripheral nerves) or non-neurological systems (e.g., muscle, heart, and pancreas) (1, 2). The number of ARCA genes is continuously expanding, extending far above >100 genes, and the first ARCAs now come into reach of targeted treatment options (2).

Disease registries have been important for identification, characterization, and aggregation of rare neurological diseases. However, the real-world quantitative and qualitative evidence in registries and registry-based natural history and outcome measure studies have now also become a precious source for planning of treatment trials and modeling trial designs and endpoints, thereby complementing the knowledge available from preclinical studies and clinical trials (3). The *ARCA Registry* was launched in 2013 in order to apply this concept to the field of ARCAs, and it remains the only multicenter registry fully dedicated to ARCAs and early-onset ataxias (EOAs), which are known to be enriched but not exclusive for ARCAs (1, 2). The overarching goal of the *ARCA Registry* is to become a key facilitator enabling trial readiness by

- providing an easily accessible, web-based, good clinical practice (GCP)-conforming, and general data protection regulation (GDPR)-compliant multicenter multi-trial registry infrastructure platform as a backbone for global trial-readiness efforts in ARCAs;
- building cohorts of sufficient size for trial-readiness studies and upcoming treatment trials through aggregating ARCA patients in an accessible, standardized, multicenter fashion around the world;
- characterizing the phenotypic spectra for ARCAs, which will inform treatment trial design and especially outcome selection for future treatment trials;
- collecting real-world natural history data for ARCAs acquired during daily clinical life across a large range of centers across the world, thereby informing design, planning, and modeling of treatment trials; and
- providing a continuous database backbone for trial-readiness ataxia consortia around the world, e.g., the German DZNE ARCA-EOA network (4), the PREPARE consortium (5), PROSPAX (6), and ARCA GLOBAL (7).

In this overview, we will describe the main methodological features and assets of the *ARCA Registry*, with examples on how it is already being utilized to improve trial readiness in the field of ARCAs, including its current use by multiple research networks. It will also illustrate the registry's potential for expansion to other partners worldwide to promote trial readiness for ARCAs.

## A Good Clinical Practice- and General Data Protection Regulation-Compliant Global Web-Based Registry: Data Capture, Data Access, and Data Sharing

**Figure 1:**
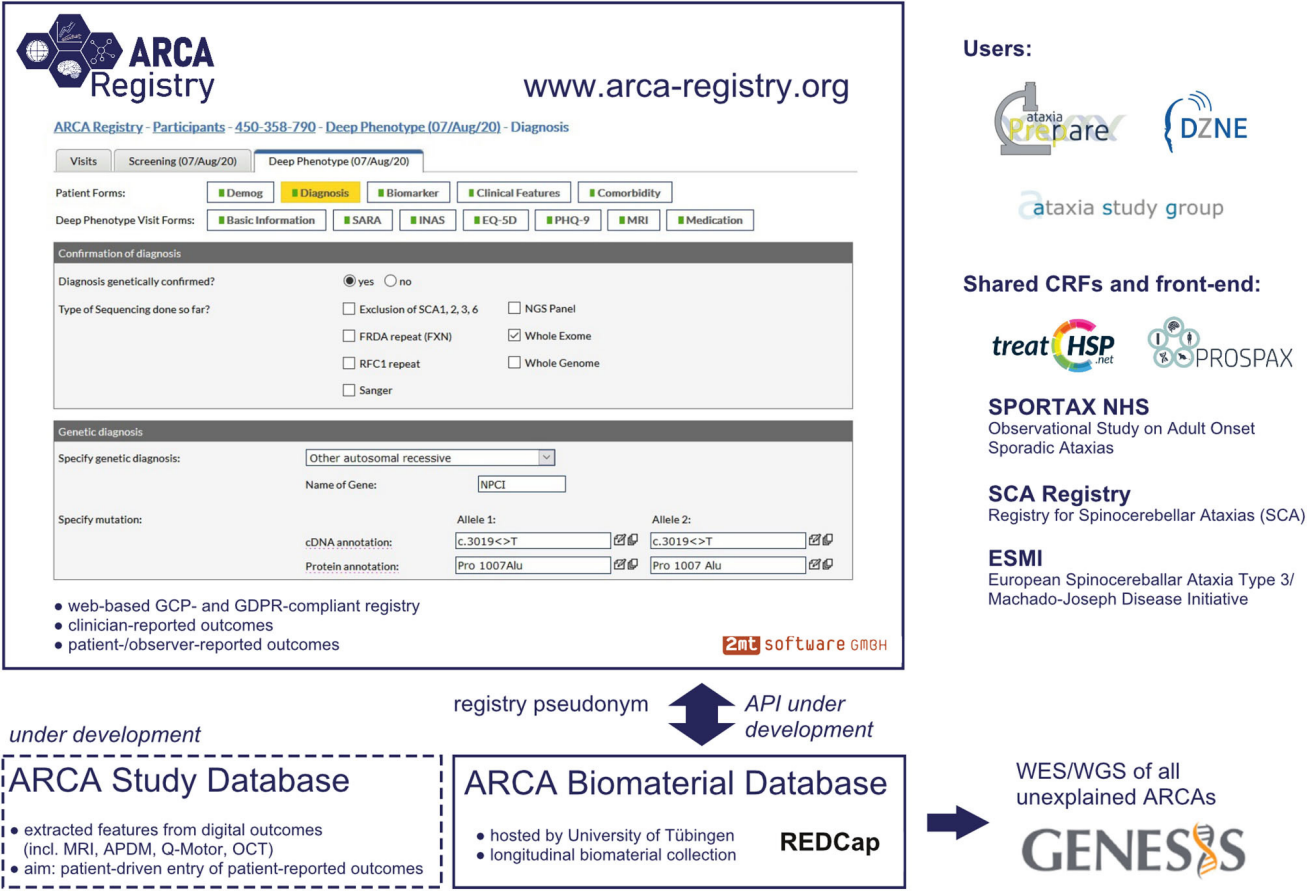
Platform and database infrastructure of the ARCA Registry. Graphical user interface of the web-based ARCA Registry, with display of a representative electronic case report form (eCRF) and embedding in the larger database infrastructure. The front-end of the software and the main core of eCRFs are shared with other major ataxia and rare disease registries (e.g., the HSP registry used by the TreatHSP network), making the ARCA Registry user friendly and convenient for joint analysis of data across genetic ataxias, sporadic ataxias, and hereditary spastic paraplegia (HSP) registries.

The *ARCA Registry* is built on WebSpirit (2mt Software, Ulm, Germany), a web-based electronic data-capture system currently used in a variety of national and international medical research consortia (Figure 1). The web-based implementation allows direct access by registered clinicians and study teams from any computer worldwide, as required for easy access in a global multicenter setting. The fact that it uses the same technical registry platform (WebSpirit) as one of the largest autosomal dominant ataxia registries, namely, the spinocerebellar ataxia (SCA)/ESMI registry (8, 9) and SCA Global (10), as well as the large sporadic ataxia registry (SPORTAX) (11, 12) and the Hereditary Spastic Paraplegia (HSP) Registry, allows for cross talk and joint analysis not only across the manifold ARCAs captured in the ARCA Registry itself but also with SCAs, sporadic ataxias, and even HSPs. This is further facilitated by aligning all key electronic case report forms (eCRFs) between these major ataxia registries. The registry platform is GCP-compliant: an audit trail is maintained to track changes to recorded data, a detailed rights and role management system limits access to entered data for each individual system user, and quality assurance is supported by an integrated online-monitoring system. Moreover, it is fully compliant with the European Union GDPR based on the following features: use of unique pseudonyms generated with a secure one-way hash function to restrict the use of personally identifiable data to local sites; separation of processing activities through assignment of user roles (e.g., data entry, monitoring, and data management) and restriction of access to data; record and transfer only of pseudonymized data; all access to data through encrypted connections; and servers located within the EU. Participating sites maintain access to their data entered in the *ARCA Registry*, with the possibility to easily export and systematically analyze locally aggregated datasets. Access to full multisite datasets is provided for specific projects upon request by a standardized project template and provided to the project submitter after evaluation of the request.

The physician-reported multidomain datasets in the *ARCA Registry* are the core of a larger systematic multi-component ARCA database cluster (Figure 1). For longitudinal collection of biomaterials, the *ARCA Registry* is linked to an *ARCA biomaterial database* built on REDCap. To facilitate whole-exome and whole-genome sequencing in all patients with unsolved ARCA, the ARCA Registry is moreover linked to next-generation sequencing (NGS) data on the genomics research platform GENESIS (13, 14). GENESIS is a user-friendly collaborative cloud-based analysis and matchmaking platform that encompasses the largest ataxia NGS dataset collection worldwide (>2,000 ataxia NGS datasets), aggregated via the PREPARE consortium (*PREPARE-GENESIS*) (see below). While the ARCA Registry and the GENESIS platform are two distinct databases, subjects from the registry are linked to the GENESIS platform via an ID generated by the ARCA biomaterial database. Ongoing developments of this multi-component ARCA database cluster will include an *ARCA multi-study database* as a repository for features of digital outcomes such as magnetic resonance imaging (MRI), digital-motor sensors (APDM, Q-Motor), and optical coherence tomography and for patient-driven entry of patient-reported outcome measures (PROMs).

## Capturing Phenotypic Spectra, Phenotypic Evolution, and Disease Progression of Autosomal Recessive Cerebellar Ataxias: The Electronic Case Report Forms

The eCRFs of the *ARCA Registry* are designed to characterize the clinical heterogeneity of phenotypic spectra and natural history phenotypic evolution of ARCAs, thus helping in the selection of outcomes and planning of upcoming treatment trials (sample size calculation, trial duration, etc.) as well-modeling of trial endpoints and treatment effects. Different degrees of eCRFs details—characterized as “core,” extended,” and “optional” datasets—allow data capture tailored to the participating site's variable interests and resources (Table 1). In brief, the eCRFs include clinical scales and composite measures, clinician-reported outcome measure and PROMs, biomarker outcomes, and quantitative performance measures:

- The core dataset in the *ARCA Registry* comprises demographic data (with ethnic background), genetic diagnosis (with types of sequencing performed), different scores to measure disease severity like the Friedreich Ataxia Rating Scale (FARS) Functional Stage (15), the Scale for the Assessment and Rating of Ataxia (SARA) (16), systematic phenotyping using the Inventory of Non-Ataxia Signs (INAS) (17) with customized amendments (e.g., bradykinesia, ptosis, or the head impulse test), the presence and onset of typical clinical ARCA features (e.g., ataxia, epilepsy, cognitive impairment, and diabetes), ARCA biomarkers (serum and neurophysiology), and relevant comorbidities (including alcohol intake).
- The extended dataset adds questionnaires on health status and depression (EQ-5D and PHQ-9) (18, 19), disease-relevant medication and treatment effects, and a summary of MRI findings.
- Optional datasets include the possibility to report pediatric features (e.g., pregnancy and birth or developmental milestones), and the ARSACS Disease Severity Index as a disease-specific outcome measure (20).

**Table 1:** Case report forms in the ARCA Registry.

**Case report form**	**Items/description**	**Dataset**
Demographics	Sex, year of birth, dexterity, ethnic background, consanguinity, siblings	Core
Diagnosis	Genetic diagnosis, type of sequencing so far, mutation/repeats (optional)	Core
Biomarkers	Biosampling for research, biochemical markers (e.g., AFP and Vit E), neurophysiology (e.g., NCS and MEP)	Core
Clinical features	Onset, course (progressive, episodic), multisystemic involvement (e.g., eyes, epilepsy, diabetes, heart, and kidney), cognition, behavior, and mainstream school	Core
Comorbidity	Alcohol, CNS/PNS unrelated to ARCA, psychiatric, and review of systems; with possible contribution to impairment	Core
SARA	Scale for the Assessment and Rating of Ataxia	Core
INAS	Inventory of Non-Ataxia Signs	Core
FARS Stage	functional staging, mobility milestones (e.g., cane, walker, and wheelchair)	Core (since 2020)
PGI-C	Patient's Global Impression of Change since last visit	Core (since 2021)
EQ-5D/EQ-5D-Y	Self-rated assessment of health status	Extended
PHQ-9	Patient health questionnaire on depression and anxiety	Extended
MRI	Summary of imaging features (e.g., atrophy and signal abnormalities)	Extended
Medication	Disease-specific; generic name, dose, target symptom, and outcome (optional)	Extended
FARS ADL	Activities of daily living	Extended (since 2021)
Pediatric features	Pregnancy, gestation, weight, head circumference, and development/walking	Optional
ARSACS DSI	Disease severity index for ARSACS	Optional

*AFP, alpha-fetoprotein; MEP, motor-evoked potential; NCS, nerve conduction studies; CNS, central nervous system; PNS, peripheral nervous system*.

The Patient's Global Impression of Change (PGI-C; core dataset) (21, 22) and the FARS Activity of Daily Living (ADL; extended datasets) (15) have recently been implemented as “anchor measures,” i.e., measures reflecting the patient's subjective experience of disease progression and functional impairment, which serve as reference measures helping to evaluate the significance of changes and effect sizes observed for the longitudinal clinical and biomarker data in the registry.

## Cross-Continental Multicenter Capture Around the World: The Contributing Centers

**Figure 2:**
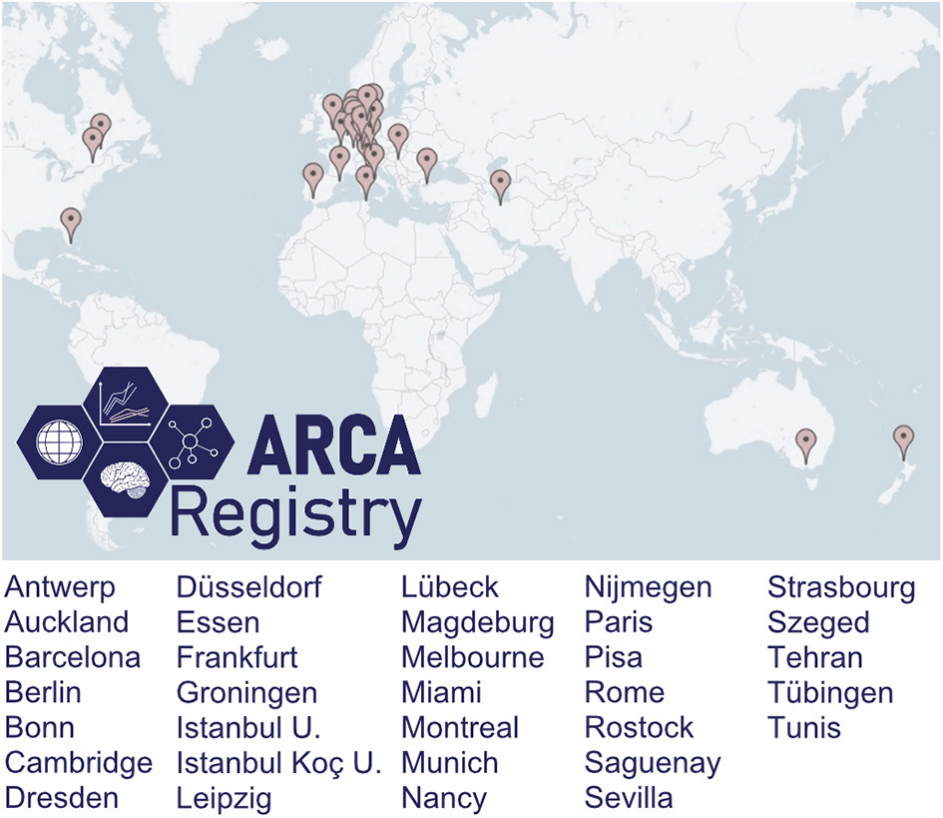
Contributing centers. The ARCA Registry is a growing, cross-continental multicenter registry, with more than 30 contributing sites in 15 countries by the end of 2020.

The *ARCA Registry* captures ARCAs from centers around the world (Figure 2). While initially mainly capturing centers from countries across Europe, the scope of the *ARCA Registry* has continuously grown in the last 5 years currently to now more than 30 sites from 15 countries. The registry has an active strategy to recruit centers from underrepresented countries to strengthen its global representation of ARCAs, regarding both disease prevalence and variable genetic/ethnic backgrounds. Participation is possible upon request. Minimum requirements are the commitment to contribute CRFs of at least the basic phenotype (see above) and to aim for longitudinal follow-ups.

## Current Data in the ARCA Registry: A Descriptive Overview of 800 Patients and 1,500 Visits

**Figure 3:**
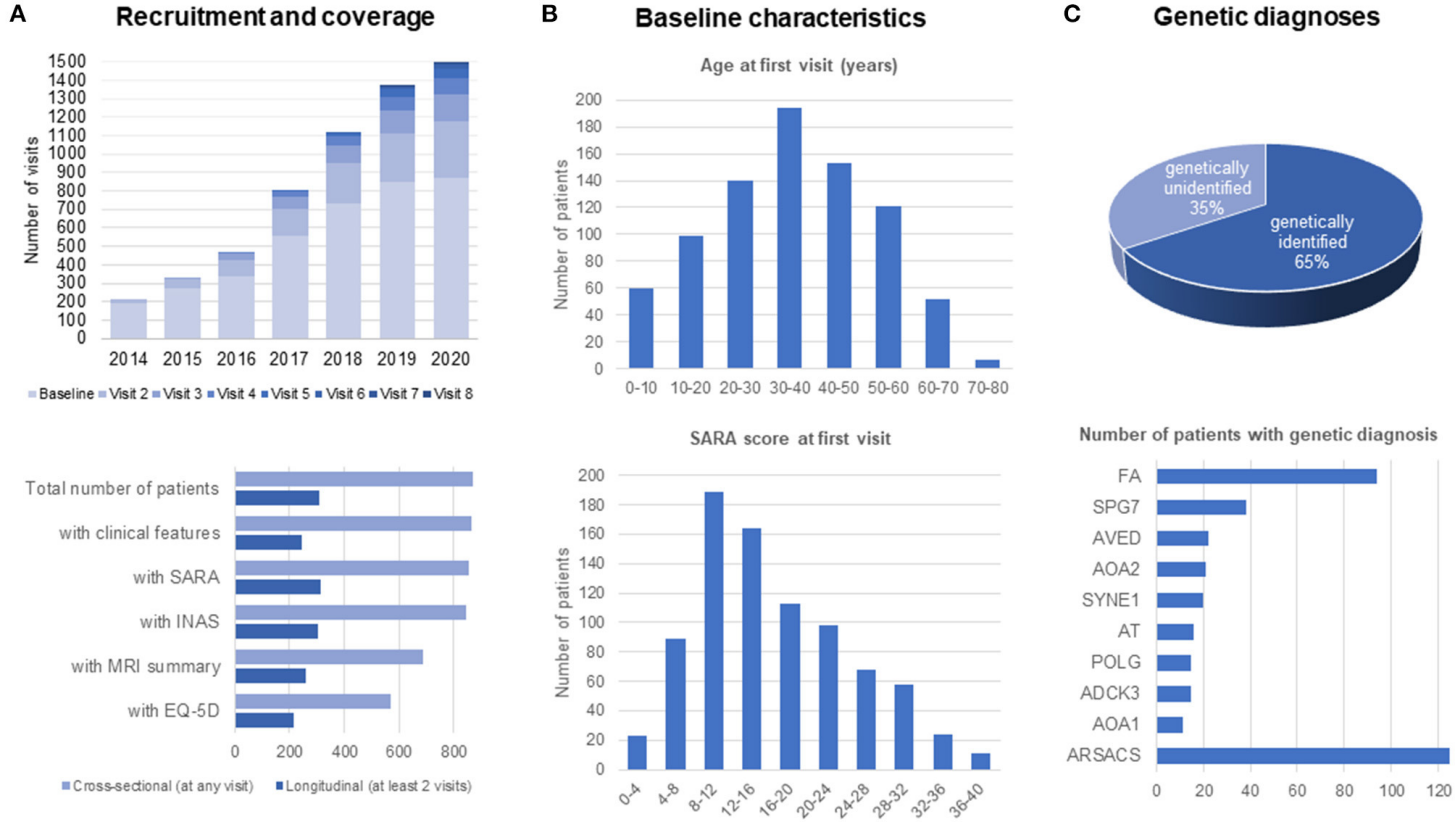
Recruitment and patient characteristics in the ARCA Registry. **(A)** Recruitment and coverage. Recruitment of patients is continuous since 2013, reaching more than 800 patients with up to eight longitudinal assessments by the end of 2020. Longitudinal data with at least two assessments are available in about 200–300 patients. **(B)** Baseline characteristics of patients. Patients in the ARCA Registry span the full range of ages and disease stages. **(C)** Genetic diagnoses in the ARCA Registry, with a list of the 10 most frequent genetic causes.

From its foundation in 2013 until now, more than 800 patients with almost 1,500 visits have been recruited to the *ARCA Registry*. In the past 5 years, there has been a considerable increase in longitudinal data, currently reaching up to eight annual follow-up visits in the first patients (Figure 3A). Follow-up core datasets including SARA or INAS from at least two visits and 13 sites are available in >300 patients. Follow-up extended datasets such as MRI summary data or the self-rated assessment of health status by EQ-5D from at least two visits are available from >200 patients. In addition to its longitudinal coverage, the *ARCA Registry* also captures patients with a broad range of ages and disease stages (Figure 3B). While—in keeping with the early onset of ARCAs (1, 2) −60% of patients have symptom onset before 40 years of age, 40% of patients have later onset, up to 80 years of age. Ataxia severity at baseline visits have been recorded as mild (SARA: ≤8), moderate (8–16), and severe (>16) in 16, 41, and 43% of patients, respectively. Sixty-five percent of patients have an established genetic diagnosis. The most frequent diagnoses in the *ARCA Registry* are ARSACS (~120 patients, 14%), Friedreich ataxia (~90 patients, 11%), and SPG7 (~40 patients, 4%; see Figure 3C for the 10 most frequent ARCAs). Except for the enrichment of ARSACS—which is an overrepresentation due to the major contribution of participating sites in Quebec—the *ARCA Registry* provides prevalence data that are generally consistent with expectations from the literature (1, 23). Patients in the ARCA Registry who do not have a genetic diagnosis yet (currently 35%) are included in a coordinated NGS effort on a continuous basis to make a diagnosis or to identify novel genes, via the PREPARE-GENESIS platform (see above).

The *ARCA Registry* with its phenotypic and longitudinal data has enabled large clinico-genetic cohort studies to delineate the phenotypic spectrum and longitudinal disease progression of major and novel ARCAs. It thus fulfills the requirements of primary datasets that can be used to describe natural history progression models and plan treatment trials in almost all of the most frequent ARCAs, including pharmacometric modeling of outcome measures and treatment effects. For example, for *RFC1*-ataxia, it has helped to reveal multisystemic phenotypes mimicking cerebellar type multiple system atrophy and progressive supranuclear palsy, and hyperkinetic movement disorders such as chorea and dystonia, and provided first sample size calculations based on longitudinal SARA assessments (24). For *COQ8A/ADCK3-*ataxia, the *ARCA Registry* facilitated the delineation of clinico-genetic associations, and the longitudinal analysis of SARA scores has provided the first systematic, group-based evidence for a possible treatment effect of coenzyme Q10 (25). Similarly, the *ARCA Registry* has enabled the natural history of *POLG*-related ataxia to be documented through longitudinal SARA and INAS assessments (26). The systematic assessment of patients with as-yet-unknown genetic molecular diagnoses means that—when the underlying gene is discovered—there are already established longitudinal progression data, as exemplified for patients found to carry pathogenic variants in the novel ARCA gene *PRDX3* (27).

## Meeting Criteria of The European Rare Disease Registry Infrastructure and of the European Medicines Agency Guidelines for Registry-Based Studies

European authorities including the European Medicines Agency (EMA) have highlighted the potential of disease registries to provide real-world evidence that can complement preclinical, clinical, and even post-marketing data especially for rare diseases (3). At the same time, however, they have also put forward clear standards for data collection and quality criteria for disease registries that aim to meet this goal.

### European Medicines Agency Registry Standards for Data Collection

The *ARCA Registry* captures the key EMA data elements (3), including administrative information (e.g., site, contact dates, registry entry and exit dates, and reason for registry exit), patient data (e.g., age, sex, and alcohol as lifestyle factor), disease features (including diagnosis, disease duration, severity/staging, genetic information, and biochemical tests if appropriate), relevant comorbidities, and disease-related or relevant concomitant medical treatment (Table 2). The *ARCA Registry* is also enrolled in the European Directory of Registries of the European Rare Disease Registry Infrastructure (ERDRI.dor). As a constituent registry of the evolving Rare Neurological Disease Registry of the European Research Network on Rare Neurological Diseases (ERN-RND) (28), the *ARCA Registry* will provide the common data elements defined by the ERDRI (29), which adds more systematic coding of diagnosis (Orpha code), genetic diagnosis (HGVS) or phenotype (HPO), resources for research (e.g., biosampling and link to biobank), and a classification of disability (Table 3). As a *cross-disease database*, the ERN-RND registry collects general information on demographics, genetics, and phenotype, which allows the identification of centers that look after specific (and often genetically defined) patient groups. By contrast, as a *disease-group specific* database, the *ARCA Registry* collects complementary in-depth data to enable trial readiness in recessive ataxias. Both registries are well-interconnected, as the common ERN dataset can be easily extracted from the ARCA Registry and imported into the ERN-RND registry. This link with the ERN-RND registry also adopts the FAIR principles in the *ARCA Registry*, ensuring that its data are *f* indable, *a*ccessible, *i*nteroperable, and *r*eusable between countries (30).

**Table 2:** Implementation of EMA guidelines on patient registries.

**Data element**	**Data items**	**ARCA registry**
Administrative information	Name of center	✓
	Availability of informed consent	✓
	Registry entry date	✓
	Registry exit date and circumstances	✓
	Dates of encounters	✓
Patient data	Age or birthdate	✓
	Gender	✓
	Lifestyle factors (alcohol, smoking, employment)	✓
Disease characteristics	Diagnosis	✓
	Date of clinical diagnosis or first consultation	✓
	Genomic information	✓
	Severity/stage	✓
	Milestones/outcomes/functional status	✓
Comorbidities	Relevant comorbidities (past/current)	✓
Disease-related and relevant concomitant treatments	Substance	✓
	Dose	✓
	Start date	(✓) ^*^
	End date	(✓) ^*^
	Route	✓
	Schedule	**×**
	Brand name	**×**
Pregnancy	Pregnancy status/outcome	**×**
PROMs	Patient-reported outcomes in clinical practice	• ^**^
Safety reporting	adverse events/reactions	• ^***^

*EMA, European Medicines Agency*.

* *Indirect assessment by longitudinal capture of current treatment*;

** *planned*;

*** *once closer to monitoring of drug treatments*.

**Table 3:** Implementation of common data elements of the EU Rare Disease platform.

**Common data element**	**Data items**	**ARCA/ERN-RND registry**
Pseudonym	Patient's pseudonym	✓
Personal information	Date of birth	(✓)^*^
	Sex at birth	✓
Patient status	Alive or dead	✓
	Date of death	✓
Care pathway	First contact with specialized center	✓
Disease history	Age at onset of first symptoms/signs	✓
	Age at diagnosis or first consultation	✓
Diagnosis	Diagnosis of rare disease in Orpha code	✓
	Genetic diagnosis in HGVS	✓
	Undiagnosed case in HPO terms	✓
Research	Agreement to be contacted for research	✓
	Consent to reuse data for other research	✓
	Biological sample available	✓
	Link to biobank where biosample is stored	✓
Disability	Classification of functioning/disability	✓

* *Restricted to year of birth*.

### European Medicines Agency Standards for Data Quality

In line with the EMA standards of data quality (3), the *ARCA Registry* aims for consistency, completeness, accuracy, and timeliness. Consistency of data is facilitated by common standardized eCRFs, by clearly defined variables and selection of questions with binary outcomes, and by the implementation of clinical scales with high interrater reliability, especially SARA, INAS, and FARS stage or ADL (15, 16, 31). Completeness of data in core datasets is automatically checked online as the first step of a continuous database-embedded monitoring process; this resulted in 95% (e.g., for clinical features of ARCAs) to 99% (e.g., for SARA or INAS) CRF completion rate. Following automated online control of data plausibility and consistency within and between different CRFs at the time of data entry, accuracy of data is afterwards controlled offline in the second monitoring step. Recruitment numbers including availability and completeness are regularly disseminated in systematic, standardized reports of networks that use the *ARCA Registry*.

## Networks Using the ARCA Registry as Their Infrastructure Backbone

The ARCA Registry is being used not only by more than 30 single sites but also by several leading ARCA networks in Europe and worldwide.

### German Autosomal Recessive Cerebellar Ataxias/Early-Onset Ataxia Network

The German network on ARCAs and EOAs, launched in 2013 by the German Center for Neurodegenerative Diseases (DZNE), comprises five major German ataxia sites (Tübingen, Bonn, Munich, Magdeburg, and Rostock). The network has established the first version of the ARCA Registry, which was co-hosted by the Ataxia Study Group. Every effort was made to ensure that the Registry is fully aligned in its data fields and database system with other major SCA and sporadic ataxia registries (32), likewise hosted by the Ataxia Study Group. Since then, the German ARCA/EOA network has contributed >400 subjects to the ARCA Registry and provides monthly reports on its recruitments to the ARCA Registry.

## PREPARE

PREPARE (Preparing for therapies in autosomal recessive ataxias) was launched in 2016 as an EU-funded (E-RARE JTC 2015) rare disease network; it was one of the first dedicated ARCA trial-readiness networks. Utilizing the complementary expertise from many ARCA centers and international ARCA networks, it was established to facilitate all crucial translational steps from genetic profiling (including discovery of new genes) to standardized preclinical trials, developing FDA-compliant outcome measures, and registry-inventoried transnational trial-ready cohorts, hereby fully building on the ARCA Registry as its backbone. The network began with seven centers from across Europe and Canada and has now expanded to >13 centers including centers in Turkey, Iran, and New Zealand. Using the extended site network and longitudinal dataset provided by the ARCA Registry, PREPARE has run phenotype and natural history studies on several ARCAs, e.g., RFC1 (24), COQ8A/ADCK3 (25), RFC1, POLG (26), and PRDX3 (27).

## Prospax

The network PROSPAX (An integrated multimodal PROgression chart in SPastic atAXias), launched in 2020 and funded by the European Joint Program on Rare Diseases (EJP RD), will establish a paradigmatic integrated trial-ready model of disease progression and mechanistic evolution in spastic ataxias. It hereby builds on a rigorous trial-like multicenter natural history center study on the two flagship recessive ataxias ARSACS and SPG7, combining longitudinal clinician- and patient-reported digital and molecular outcomes for these spastic ARCAs. It unites all major European ARCA and HSP networks and includes Canadian ARCA centers (>7 centers) to run this transatlantic natural history study. PROSPAX hereby utilizes a “spin-off” study registry version, which directly builds on the ARCA Registry, with fully compatible pseudonymization procedure and eCRFs, and where datasets will be integrated into the ARCA Registry (and equally the HSP registry) at the end of the study. PROSPAX also draws on all the other components of the multi-component ARCA database cluster described above (ARCA biomaterial database, GENESIS, and ARCA multi-study database). By sharing the same core eCRFs and front-end with the HSP Registry used by the TreatHSP network (33), this spin-off version of the ARCA Registry enables direct cross talk with the HSP registry and joint analysis with HSPs, which is of high importance given the large genetic, molecular, and clinical overlap between ataxias and HSPs (34).

### ARCA Global

ARCA GLOBAL and its sister platform SCA GLOBAL together comprise the Ataxia Global Initiative (AGI). The AGI presents a worldwide multi-stakeholder (academia, industry, and patient organizations) platform coordinating and preparing all necessary steps for trial readiness in autosomal-dominant (SCA GLOBAL) and autosomal recessive (ARCA GLOBAL) ataxias (7, 10). With establishing trial-ready cohorts and cross-center harmonized clinician-reported outcome measures and PROMs as one of its key tasks, the AGI uses the ARCA Registry as one of its key registries. This reflects the fact that the ARCA Registry already captures all outcome measures that were stipulated by the AGI as the common core set of clinical outcome measures to be used by ataxia centers worldwide. Moreover, the AGI builds on the ARCA Registry as one of its major trial-readiness resources, as this registry readily allows data-download and dataset preparation for further workup, e.g., by the Critical Path to Therapeutics for the Ataxias (CPTA) consortium of the Critical Path Institute (C-Path), which aims to prepare regulatory approval by the FDA and the EMA for clinical endpoints in genetic ataxias.

In addition, the SPATAX network, which includes all types (i.e., not only autosomal recessive) of ataxias and HSPs, has contributed subsets of data to the ARCA Registry.

## Limitations and Outlook

The ARCA Registry faces several limitations and open challenges that remain be to be addressed:

- The sustainability of the ARCA Registry depends on strong commitment by the contributing centers as well as project-based funding, with fluctuations in patient recruitment, participating sites, and monitoring performance. Technical improvements and new software implementations often come along with variable latencies. Even the “core dataset” may exceed the possibilities of a clinical appointment in many ataxia centers, but further minimization of the core dataset would need to be carefully weighed against the minimal required data necessary to really help in preparing trial readiness as well as against registry standards put forward by public authorities. The timeliness of monitoring is governed by batch monitoring of each site by site, which provides the opportunity for focused local revision of data and files but also leads to periodic delays for those sites that had just been monitored.
- Patients with the same genetically defined ARCA are still dispersed in different ataxia registries, e.g., because of identification of novel autosomal recessive genes in sporadic late-onset ataxia patients (e.g., RFC1) who have so far been collected in a sporadic ataxia registry (e.g., the SPORTAX registry).
- The ARCA Registry does not cover all aspects of each ARCA disease, or may cover it with measures that are too broad for clinical trial design in a specific ARCA disease. While the global phenotype or the progression of ataxia as measured by the SARA score is assessed, more fine-grained motor (e.g., walking speed) and especially a larger array of non-motor features (e.g., the cerebellar cognitive-affective syndrome) are not captured. Moreover, selected eCRFs and non-ataxia scales like the INAS were primarily implemented to systematically capture disease phenotypes but might show less responsiveness to change. Thus, for capturing the natural history of certain ARCAs, the ARCA Registry might need to be complemented by additional eCRFs. Registry spin-offs such as PROSPAX registry, however, exemplify that the registry infrastructure can indeed be readily adapted to meet the needs of such natural history studies.
- Finally, recruitment into the ARCA Registry is still biased toward patients from Europe or of European descent, which leads to an underrepresentation of ARCAs with other ethnic/genetic as well as sociocultural backgrounds. Structural disadvantages (especially availability of local person and funding resources for data-entry) and language barriers may hamper a more global dissemination of the ARCA Registry. The eCRFs, the registry software, and templates for an application to a local institutional review board are all available in English language, but additional translations and country-specific adaptations may help to increase the scope.

## Conclusions

The ARCA Registry has (i) enabled the harmonization of clinical outcomes across ataxia centers around the world; (ii) has demonstrated its capacity to act as a centralized database for genotype–phenotype and natural history studies in the >100 ARCAs, already exemplified for *COQ8A*-, *RFC1*-, and *POLG*-related ataxias; and (iii) aggregates the necessary large-scale longitudinal progression datasets for calculating sample sizes, modeling trial designs and randomization procedures, and running pharmacometric models simulating treatment effect sizes for anticipated clinical trials. Given its adoption by many international ARCA sites and networks; its GCP, GDPR, and EMA compliance; its web-based data capture; and its connections to a constantly growing multi-component ARCA database cluster, the ARCA Registry is well-placed to become a global trial-readiness registry for ARCAs.

## Co-Investigators of the *PREPARE* Consortium

Sarah Doss, Department of Neurology, Charité University Medicine, Berlin, Germany; Jun-Suk Kang, Department of Neurology, University Hospital, Frankfurt, Germany; Ivana Ricca, IRCCS Fondazione Stella Maris, Pisa, Italy; S. Ben Sassi, Neurology Department, National Institute of Neurology, Tunis, Tunisia; Laszlo Szpisjak, Interdisciplinary Excellence Centre, Department of Neurology, Faculty of Medicine, Albert Szent-Györgyi Clinical Center, University of Szeged, Szeged, Hungary; Andreas Thieme, Department of Neurology, Essen University Hospital, University of Duisburg-Essen, Essen, Germany

## Author Contributions

AT and MS have designed and conceptualized the review and analyzed the data. All authors have contributed data to the ARCA Registry and/or drafted or revised the manuscript for intellectual content, contributed to the article, and approved the submitted version.

## Conflict of Interest

MW is the director of 2mt Software GmbH. The remaining authors declare that the research was conducted in the absence of any commercial or financial relationships that could be construed as a potential conflict of interest. The reviewer JT declared a shared affiliation, with no collaboration, with one of the authors AM to the handling Editor.

## Abbreviations

ADLs: activities of daily living
AGI: Ataxia Global Initiative
ARCA: autosomal recessive cerebellar ataxia
DZNE: German Center for Neurodegenerative Diseases
ESMI: European Spinocerebellar Ataxia Type 3/Machado-Joseph Disease Initiative
eCRF: electronic case report form
EMA: European Medicines Agency
EOA: early-onset ataxia
ERDRI: European Rare Disease Registry Infrastructure
ERN-RND: European Reference Network for Rare Neurological Diseases
FARS: Friedreich Ataxia Rating Scale
GCP: good clinical practice
GDPR: general data protection regulation
HSP: hereditary spastic paraplegia
INAS: Inventory of Non-Ataxia Signs
MRI: magnetic resonance imaging
NGS: next-generation sequencing
PGI-C: Patient's Global Impression of Change
PHQ-9: patient health questionnaire on depression and anxiety
PROM: patient-reported outcome measure
SARA: Scale for the Assessment and Rating of Ataxia
SPORTAX: Consortium “Sporadic Degenerative Ataxia with Adult Onset”
PREPARE: Consortium “Preparing for therapies in autosomal recessive ataxias”
PROSPAX: Consortium “An integrated multimodal PROgression chart in SPastic atAXias”
SCA: spinocerebellar ataxia.
